# Supplementary material for: Are carbon emissions trading and green financial instruments synergistic? -Comprehensive quantitative research based on content analysis
Source: PLoS One. 2024 Mar 7;19(3):e0298601. doi: 10.1371/journal.pone.0298601 (PMC10919601; doi:10.1371/journal.pone.0298601)
Supplement: S1 Data — (ZIP) [file pone.0298601.s001.zip › Data/地级市Z_F工作报告.zip!/╣π╢1⁄2/╟σ╘╢╩╨/└┤╫╘╣1⁄2╓┌║┼í╛┬φ┐╦╩2╛▌═°í┐_2007.docx]

各位代表：

我代表市人民政府向大会作政府工作报告，请予审议，并请政协各位委员和其他列席人员提出意见。

本届政府工作回顾

市四届人大一次会议以来，在省委、省政府和市委的正确领导下，在市人民代表大会及其常务委员会和市政协的监督支持下，在历届政府奠定良好发展的基础上，本届政府始终坚持继承、发展和创新的一贯思想，大力发扬历届班子真抓实干、艰苦创业、实事求是和奋发进取的精神，高举邓小平理论和“三个代表”重要思想伟大旗帜，坚持以科学发展观统领经济社会发展全局，认真贯彻落实党的十六大和省第九次党代会以来的方针政策，按照市第四次党代会的部署，围绕“三年打基础，五年上台阶，十年翻两番，二十年全面建成小康”的总目标，大力实施“三化一园”发展战略，经受住非典型肺炎和高致病性禽流感疫情的严峻考验，成功战胜严重干旱、洪涝等自然灾害，有效解决了发展中遇到的突出困难和问题，提前一年基本完成了市四届人大一次会议确定的各项任务，顺利实现了市四届人大四次会议确定的2006年各项预期目标，实现了“十一五”时期的良好开局。四年来，我市经济社会呈现出大发展、大跨越的欣欣向荣局面。从2004年起连续三年，在全省公布的八项主要经济指标中，我市取得了四项以上主要经济指标增长全省“三连冠”的历史最辉煌成绩，创造了令人瞩目的“清远现象”。

一、 经济持续高速增长，综合实力显著增强

据快报统计，2006年全市实现GDP411亿元，比上年增长26.5%，高出全省12.5个百分点，比2002年增长1.2倍。人均GDP突破1400美元，比上年增长22.3%，比2002年增长1倍。

工业经济高速增长。2006年全部工业总产值达到660亿元，比上年增长60%，比2002年增长4.5倍。规模工业实现增加值148.4亿元，比上年增长71.3%，高出全省增幅51个百分点，比2002年增长4.4倍。工业化成为我市经济增长的重要引擎。工业化率由2002年的18%上升到 2006年的41%，对经济增长的贡献率由17.8%大幅提升到73%。

第三产业快速发展。加大旅游基础设施建设投入，旅游环境进一步优化，2006年，全市游客突破1100万人次，旅游收入35.9亿元，分别比2002年增长1.64倍和1.86倍。着力扩大消费需求，社会消费品零售总额2006年达到159亿元，比上年增长22.8%，比2002年增长82.5%。大力发展房地产业，2006年房地产开发投资达39.3亿元，增长45.2%，比2002年增长2.6倍，四年累计房地产开发投入资金97.3亿元，商品房竣工面积503万平方米。涌现出一批设施齐全、环境优美的大型高档楼盘。现代服务产业迅速成长。金融保险、咨询、中介、现代物流、计算机服务等新兴服务业发展迅猛。第三产业实现增加值145.6亿元，比上年增长15%，比2002年增长93%，增长速度位居全省前列。

民营经济迅速壮大。全市注册个体工商经营户7.18万户，注册资金23.9亿元，比2002年增长1.4倍。民营企业4882家，比2002年增长2.9倍；注册资金98.7亿元，比2002年增长5.6倍。2006年，民营经济完成投资154.6亿元，占全市固定资产投资总额的47%。规模以上民营工业企业213家，比2002年增长2.04倍；资产总计140亿元，比2002年增长2.6倍；实现利税9亿元，比2002年增长3.4倍；实现工业产值265.5亿元，比2002年增长12.3倍。民营工业产值占规模工业产值的份额达到42.8%，成为我市经济增长的重要生力军。

县域经济加速发展。南部地区继续领跑全市经济，清城区、清新县和佛冈县GDP分别比2002年增长1.84倍、1.37倍和1.33倍；中部地区快速崛起，英德市GDP比2002年增长1.08倍；北部地区迅速发展，连州市、阳山县、连南瑶族自治县和连山壮族瑶族自治县GDP分别比2002年增长87%、65%、59%和52%。镇域经济迅速壮大，涌现出了一大批工业强镇，2006年，有13个镇（街）的工业总产值达10亿元以上，其中有3个镇达50亿元以上，清城区龙塘镇突破100亿元。

综合实力显著增强。随着经济持续高速增长，我市经济总量已领先于全省山区市，多项指标在全省21个地级市中的排位明显提升。2006年，GDP列全省第14位，与2002年相比提升了4位；全部工业总产值列全省第12位，提升了6位；固定资产投资总额突破300亿元，列全省第7位，提升了9位；外贸出口总额12亿美元，列全省第13位，提升了4位；实际利用外资总额3亿美元，列全省第10位，提升了6位；地方财政一般预算收入列全省第14位，提升了2位。

二、经济结构不断优化，经济效益显著提高

经济结构明显优化。着力调整农业产业结构，大力发展二、三产业，三次产业结构由2002年的35.8:22.7:41.5，调整为2006年的18.2:46.4:35.4。工业经济突飞猛进，初步形成了建材陶瓷、电力、机械装备、金属制品、纺织服装、制鞋皮革、电子家电、农副产品加工、化学医药、废旧金属材料回收等十大产业体系。现代农业、特色农业、效益农业和生态农业发展较快，粮经作物比例调整为47：53，实现粮经作物比例大掉头。种养比例为62：38，养殖业比重比2002年上升了5个百分点。

经济效益显著提高。2006年规模以上工业实现利润总额15亿元，比上年增长1.5倍，比2002年增长4倍；实现利税30亿元，比上年增长1倍，比2002年增长2.6倍。财税收入大幅增加，2006年，来源于清远的财政总收入44.5亿元，比上年增长34.5%，其中税收收入41.4亿元，增长38.4%；地方财政一般预算收入19.6亿元，比上年增长47.9%，比2002年增长2.2倍。节能降耗工作取得新成效，每万元规模以上工业增加值耗电量比上年下降6.5%。

物价金融形势稳定。总体物价水平平稳，经济运行处在“高增长、低通胀”的良好状态。2006年居民消费价格总水平上涨1.3%。金融运作稳健，对地方经济发展作出积极贡献。2006年末，金融机构各项存款余额470.83亿元，比上年末增长21.9%，比2002年增长 83.7%；各项贷款余额190.05亿元，比上年末增长14.6%，剔除不良资产剥离因素，实际贷款余额比2002年增长45.1%。

科技创新能力不断提高。四年新增省级高新技术企业33家，组建省级工程技术研究开发中心1家。2006年，40家高新技术企业实现工业总产值149.99亿元，出口创汇3.56亿美元。清城区源潭镇、龙塘镇和清新县飞来峡镇、山塘镇、龙颈镇以及佛冈县石角镇被认定为广东省技术创新专业镇。

品牌创建实现新突破。继荣获“中国优秀旅游城市”后，又荣获了“广东省文明城市”、“广东省双拥模范城”、“中国漂流之乡”、“中国温泉之乡”、“中国龙舟之乡”等8个区域性品牌。连南“耍歌堂”获“中国非物质文化遗产”称号。积极鼓励和推动企业创名牌。四年共获国家和省级名牌产品或著名商标31项，7个产品被评为“国家免检产品”。1个产品被评为“中国名牌产品”, 1个产品被确认为“中华老字号”品牌，8个系列产品荣获“广东省食品文化遗产”称号。3个产品获得国家地理标志产品保护。1个产品获得原产地证明商标。9类农业特产产区获中国特产之乡称号，绿色食品认证实现零突破。

三、基础设施显著改善，城市化进程明显加快

四年共完成固定资产投资798.3亿元，是建市以来至2002年15年间投资总和的1.42倍，其中基础设施建设投资254亿元。重点项目建设进展顺利，发展基础更加坚实。

交通网络不断完善。四年累计投入交通设施建设资金96亿元，提前实现省城到市区通高速公路和市到县通二级公路目标，在全省山区市率先实现县（市）到镇通等级公路。2006年末，全市公路通车里程达到1.09万公里，四年新增1670公里，其中高速公路119.4公里；每百平方公里公路密度从2002年的52.5公里提高到57公里。

信息化建设取得新进展。通信设施达到全省中等水平。2006年，全市互联网用户8.5万户，比2002年增长3倍；电话机用户222.8万部，平均两人超过一部，其中，移动电话用户158.8万户，增长1.95倍。

水利建设得到加强。四年累计投入水利建设资金30.2亿元，完成了一批城乡水利防灾减灾工程、农村饮水工程、水库除险加固工程等重点项目，加固江河堤围180.6公里，加固小型水库174座，改善灌溉面积33.7千公顷，新增除涝面积16.9千公顷，水利防灾减灾能力显著提高。

能源建设大步迈进。四年完成电力项目投资83.3亿元。完成了粤连火电厂二期、阳山火电厂等一批项目的建设改造。新增发电装机容量35万千瓦，比2002年增加5万千瓦。建成了110千伏以上输变电工程38项以及大批农城网工程,新增主变容量159.95万千伏安。2006年，全社会用电量达80亿千瓦时，比上年增长34.5%，比2002年增长2.15倍。

城镇市政基础设施建设力度加大。四年投入城市基础设施建设资金15.01亿元，市区主要道路黑底化改造基本完成。老城区东拓工程取得突破性进展，大市区框架初步奠定。城市排污、生活垃圾处理能力显著增强，各县（市）的县城中轴线、各项配套设施建设改造，以及道路美化、绿化、亮化步伐加快，中心城镇服务能力大大增强，城市形象大大提升。同时，改革城镇户口管理制度，城镇的集聚和辐射带动能力增强，以常住人口计算的城镇化水平达到40.9%，比2002年提高4.1个百分点。

四、“三农”工作进展顺利，新农村建设稳步推进

农业农村经济发展势头良好。2006年，实现农林牧渔业总产值113.3亿元，比上年增长4.7%，比2002年增长17.3%。

支农惠农政策得到落实。四年来，我市以发展农业农村经济为重点推进新农村建设，农村基础设施建设和社会事业发展取得新成绩，着力建立农民增收长效机制，解决涉及农民切身利益的突出问题成效明显。落实了各项支农惠农政策，全面免征农业税，减轻农民负担3.15亿元,人均减负100.49元；对粮农实行规模种粮直接补贴，财政新增种粮直补资金累计1338万元；扶持农业机械化专项资金1159万元；2006年财政新增综合直补资金2000万元。规范涉农收费，严格执行涉农收费公示制、农村中小学教育收费“一费制”、订阅报刊限额制、农民负担案（事）件责任追究制四项制度，农民负担明显减轻。成功举办两届农业博览会。努力提高农民素质。四年共培训农业劳动力16.3万人，转移就业39.9万人。

现代农业发展步伐加快。农业产业化组织程度不断提高，农业龙头企业带动作用不断增强。88个农业龙头企业带动农户23.6万户，增收4.87亿元，户均年增收2062元。特色农业经济区域逐渐形成，农业标准化工作稳步推进。已建立省级农业标准化示范区7个，在建3个。110家生产单位通过无公害农产品产地认定，164个产品通过无公害农产品认证。认定生产基地种植面积55.92万亩。

生态林业建设加快。地方财政用于林业支出金额3.09亿元，完成56万亩荒山绿化任务，“市肺”工程进展顺利。连山壮族瑶族自治县、清新县获“广东省林业生态县”称号，连州市、连山壮族瑶族自治县获得“全国造林绿化百佳县”称号。全市自然保护区总面积达到30.3万公顷，占全市面积的15.8%。林木绿化率70.9%，森林覆盖率提高到66.7%，分别排在全省第三和第四位。

社会主义新农村建设顺利推进。按照“一年起好步，三年打基础，五年见成效”总体工作目标和建设具有清远特色社会主义新农村的总体要求，2006年重点规划建设的80个新农村试点村建设正在顺利推进。农村危房改造、安居工程、重建家园工作取得显著成效，2966户全倒户于2007年元旦前全部搬入新居。

五、发展空间不断拓展，对外开放与区域合作水平进一步提高

积极开展对外活动。四年来，我市大力实施“走出去”战略，积极组团参加省在欧洲、美洲、日韩、澳洲及东盟等国家和地区进行的推介和经贸交流活动；每年组团参加粤港经贸交流洽谈会；认真组织在珠江三角洲、长江三角洲等地区开展经贸洽谈活动和参加省“山洽会”、“农博会”等经贸活动，不断提高对外开放与区域合作水平，清远的知名度和对外形象日益提升，成为外来投资和经贸合作的新热点。在各类经贸交流活动上，四年共签订投资贸易合同金额12亿美元。

对外经济贸易快速发展。四年累计实现外贸出口总额29.5亿美元，其中2006年实现出口总值11.83亿美元，比2002年增长4.38倍。四年来，全市新批外资项目540个，合同外资金额22亿美元，实际吸收外资金额6.35亿美元；2006年，实际吸收外资3.2亿美元，比2002年增长1.91倍。目前，我市吸收外资总量不断增大，质量不断提高，外资来源越来越广，有力地拉动了全市经济的发展。

招商引资成绩显著。坚持把招商引资作为经济工作的重中之重，创新招商引资方式方法，对外商投资吸引力显著增强，呈现出引进项目越来越大，投资金额越来越多，科技含量越来越高，对地方财力贡献越来越大的喜人局面。四年来，全市共引进项目2302个，计划总投资1673亿元，实际已投入347亿元。其中投产项目1014个，在建项目499个，筹建项目409个。现有外资企业762户，注册资本28.94亿美元。2006年，全市共引进项目622个，计划总投资471亿元，累计实际投入资金60多亿元。其中投产项目144个，在建项目189个，筹建项目289个。

六、社会事业全面进步，和谐清远建设初见成效

加强思想道德教育，倡导社会文明风尚。扎实推进“爱国、守法、诚信、明礼”现代公民教育活动，掀起学习社会主义荣辱观的热潮。创建文明城市、文明社区、文明村镇、文明单位和文明家庭等活动稳步推进。

大力发展教育事业。四年来，各级财政投入教育事业资金37.6亿元，促进了教育事业大步发展，“普九”成果得到巩固提高。创省、市一级学校80所，其中省一级学校15所。创国家级示范性高中成绩显著，清远市第一中学被确认为国家级示范性高中，英德中学、南阳中学、连州中学、清新一中等四所中学通过了初期督导验收。2006年秋季起，全市农村义务教育全部免收学杂费。适龄儿童入学率达99.3%，初中入学率达98.3%。民办教育快速发展，全市共有民办教育机构334所，在校生7.6万人。职业教育发展迅猛，创建了清远职业技术学院，引进了南华工商学院，清远市高级技工学校升格为国家级重点高级技工学校、技师学院，清远职业技术学校被评为国家级重点职业技术学校，全市中职学校在校生3.13万人，比2002年增长1.1倍。

大力发展文化体育事业。与中央电视台联合成功举办了“心连心”慰问演出，成功举办了连州国际摄影年展等系列文化活动。率先打出“生态体育”品牌，成功举办第三届市运会，圆满完成了世界杯飞碟赛、广东省第三届少数民族传统体育运动会等一系列国际、全国和全省体育赛事的承办任务。在第十二届省运动会中，共获得金牌14枚，总分964分的好成绩。文艺创作、文化遗产保护取得丰硕成果。广播影视、新闻出版和哲学社会科学事业取得新成绩。

不断加大环境保护力度。全市工业废气治理率达到92%，工业废水处理率超过90%，处理达标率达到92.8%，工业固体废物综合利用率达到69.8%。主要江河水质良好，饮用水源水质达标率为100%。空气环境质量达到国家二类区标准。积极推进循环经济和清洁生产。创建循环经济产业园区，建立能耗指标公报制度，实施节能指标责任制。推广清洁生产，建设再生资源回收体系。

加强人口和计划生育等其他工作。严格落实人口与计划生育各项政策，人口与计生工作升至全省中上水平。认真落实民族区域自治政策，以办特区的理念发展民族经济，少数民族地区经济社会快速发展。国防动员、人民防空、民兵预备役工作取得新成绩。大力开展法制各项工作，“四五”普法任务全面完成，依法行政水平有新提高。反腐倡廉和社会监督工作呈现良好发展态势。国土资源、城镇规划、统计、审计、知识产权保护、外事、侨务、对台、民族宗教、人事、妇女儿童、扶贫、移民、武装、气象、地震、档案、方志、残联、殡改等各项工作有新发展。

人民生活水平明显提高。2006年，在岗职工平均工资20705元，比上年增长13%，比2002年增长69%。农民人均纯收入3872元，比上年增长7%，比2002年增长26.8%。城乡居民储蓄存款余额达333亿元，比上年末增长21.6%。小轿车等高档消费品逐步进入百姓家庭，2006年末，全市私人小轿车保有量超过一万辆，年均增加1500辆。城镇居民人均居住面积达到26.4平方米，增加12.2平方米。人民生活水平总体上已迈向更加富裕的小康。

七、努力解决民生问题，共享改革发展成果

做好就业再就业工作。2003年以来，新增就业岗位17.5万个，全面实施农村贫困户劳务扶贫和零就业家庭就业援助制度，统筹城乡居民就业工作和加强高技能人才培养，实施“百万农村青年技能培训工程”和“退役士兵职业技能培训工程”以及有效开展残疾人、受灾群众免费技能培训。城镇登记失业率控制在3.2%以下。

社会保障工作取得新突破。社会保险由市级统筹，构建多层次养老保险体系，参保人员达90.62万人次，各项社保基金累计结余13.6亿元。非公有制企业职工纳入医保覆盖率范围，国有企业离退休人员基本养老金和下岗职工基本生活费按时足额发放。城乡最低生活保障基本实现应保尽保。低保覆盖人口已达12.9万人，占全市人口的3.2%。全面落实优抚政策。四年累计安排抚恤和社会福利救济资金5.09亿元，救济困难群众430万人次，为1.5万多名残疾人提供康复服务以及补助孤寡老人和特困复员军人、五保户、低保户。

严格执行国有企业改制职工分流安置政策。认真落实国有企业改革“三条准则”，积极实施《广东省工资支付条例》，及时调整最低工资标准，积极开展清理拖欠农民工工资及各项工程欠款，切实维护劳动者的合法权益，劳动关系稳定和谐。

加大公共卫生事业投入。四年来，各级财政投入卫生事业7.4亿元。加强了公共卫生体系建设，以市疾控中心为主的疾病预防体系建设任务基本完成。医疗救治体系建设步伐加快，应对突发公共卫生事件能力进一步提高。大力改善农村医疗卫生条件，农村卫生院“改薄”有效推进。大力推进新型农村合作医疗。2006年，全市参加新型农村合作医疗人数为200.4万人，占农业人口的66.2%。

切实加强安全生产和公共卫生安全工作。健全安全生产监管体制，以铁的手腕依法炸封关闭51对证照不全的小煤矿，工矿生产、交通、火灾等各种安全事故发生数和伤亡人数大幅下降。继续整顿和规范市场秩序，依法严厉打击商业贿赂、假冒伪劣、价格欺诈等不法行为。进一步完善食品准入管理，将食品安全准入管理延伸到农村市场。深入开展社会治安综合治理，加快社会治安防控体系建设，全面推进平安清远建设，严厉打击各种黑恶势力、严重刑事犯罪和“两抢”、“两盗”等多发性犯罪；大力整治治安重点地区和处置突出治安问题，坚决扫除“黄赌毒”、传销和变相传销及“六合彩”赌博，人民群众安全感进一步增强。

高度重视信访工作。认真调处化解各种矛盾纠纷，积极预防和妥善处置群体性事件，人民群众的合理诉求得到妥善解决，群众集体上访、越级上访事件大幅下降，有效维护了社会和谐稳定。

八、体制改革不断深化，施政能力明显提高

经济体制改革深入推进。国有企业改革初步完成，政府机构改革顺利完成，全市精简行政机构6%，精简行政编制25%；行政审批、核准事项由567项减少到192项，减幅达66.1%。乡镇撤并率达到44%。金融改革取得初步成果。卫生、教育、交通资源整合卓有成效。改革创新最大限度地解放了生产力，激发了发展潜力。

政府管理能力和服务水平不断提高。四年来，我们严格按照“行为规范、运转协调、公正透明、廉洁高效”的要求，切实转变政府职能，修改制定《政府工作规则》，认真贯彻实施《行政许可法》和《公务员法》，严格规范政府行政行为。积极实施《全面推进依法行政实施纲要》，大力推进依法行政工作，认真执行向市人大及其常委会报告工作制度，坚持向人民政协通报情况。认真办理人大议案、建议和政协提案及群众来信。积极推进行风评议，大力整顿机关作风，着力加强自身建设，依法行政水平和政府调控宏观经济能力进一步提高。

各位代表，过去的四年，是我市发展历史上不平凡的四年，是清远发展跨上新台阶、开创新局面的四年。四年的发展成就来之不易，这是省委、省政府和市委正确领导的结果，是各级人民代表大会及其常务委员会和人民政协监督支持的结果，是全市上下群策群力、艰苦奋斗的结果，是港澳台同胞、海外侨胞和社会各界关心支持的结果，是兄弟地区尤其是挂扶单位大力帮助的结果。在此，我代表市人民政府向全市广大工人、农民、知识分子、干部职工、驻清人民解放军、武警、消防官兵、人民警察和各民主党派、人民团体、社会各界人士表示崇高的敬意！向长期关心和支持清远建设的兄弟地区、香港和澳门特别行政区同胞、台湾同胞、海外侨胞和外籍友人，表示衷心的感谢！

四年的实践，使我们得到了一些启示：

第一，必须始终坚持发展为第一要务，全力推进经济社会高速发展。四年来我们始终坚持以经济建设为中心，大力推动经济持续、高速、健康发展，多项主要经济指标增幅连续三年位居全省前列。实践证明，只有抓住经济建设不放松，大力发展生产力，才能为构建和谐清远，为实现全市人民的富裕安康提供坚实的物质基础。

第二，必须始终坚持科学发展观，统筹经济社会全面、协调、可持续发展。四年来，我们在加快经济发展的同时，致力于“五个统筹”协调发展。实践证明，只有统筹发展，才能促进经济社会又好又快发展。

第三，必须始终坚持改革开放，不断增强发展的活力和动力。四年来，我们始终坚定不移地推进各项改革，扩大对外开放，促进体制机制创新，消除制约经济发展的体制性障碍，不断激发发展的内在活力。坚持把招商引资作为工作重中之重，引进国内外资本和先进技术，借助外力增强加速发展的动力，形成开放型经济格局。实践证明，坚持改革开放，是壮大工业规模和增强经济实力的重要举措，是实现跨越式发展的必由之路。

第四，必须始终坚持以人为本，关注民生。四年来，我们坚持以人为本，把解决民生问题放在发展的前提与归宿的重要位置上，坚持发展为了人民，发展成果由全体人民共享。大力推进“十项民心”工程建设，努力解决人民群众最关心、最直接、最现实的利益问题，不断提高人民群众物质文化生活水平和社会保障水平，维护社会安定有序和公平正义，确保人民群众安居乐业。实践证明，只有坚持立党为公，执政为民，实现好、维护好、发展好人民群众的根本利益，让人民群众共享发展成果，才能最大限度地调动人民群众的积极性、主动性和创造性，形成人人支持改革，人人推动发展的良好局面。

第五，必须坚持依法行政，树立法律权威。四年来，我们严格依照法定的权限和程序行使权力、履行职责、接受监督。带头学法、守法、用法，运用法律思维方式处理问题、解决矛盾，坚决摒弃以权代法、以权压法、蔑视法律的封建习气，增强了人民群众对法治的信心，有力地促进社会的和谐建设和政府的自身建设。实践证明，只有坚持依法行政，运用法律处理经济社会发展的问题，同时，辅以经济、行政和思想教育等必要手段，政府才能真正具有较高的执行力和公信力。

各位代表，今天的清远确实发生了翻天覆地的变化。清远的变化，体现在发展理念、发展道路、发展模式的“质变”,体现在经济社会发展由起步阶段进入起飞阶段的“质变”。但我们必须清醒地看到，我市经济和社会发展仍然存在不少困难和问题，主要表现在：自主创新能力弱，经济增长主要依靠资本投入和资源、能源消耗的状况尚未根本改变；发展不够平衡、不够协调、不够全面的问题依然存在，南北部发展差距拉大，城乡差距扩大的趋势还没有得到有效缓解；工业反哺农业、城市支持农村的能力不强，农村公共服务水平滞后，农民持续增收难度加大；结构性失业矛盾突出，劳动就业压力大和工业园区招工难两方面问题并存；环境状况仍有待进一步改善，经济发展与环境建设、生态保护之间的矛盾凸显，可持续发展受到严峻挑战。这些问题必须予以高度重视，并切实采取有效措施加以解决。

今后五年工作的总体要求和奋斗目标

今后五年，是我市加快发展、科学发展，全面建设小康社会的关键时期。机遇与挑战并存，机遇大于挑战。放眼世界，经济全球化和区域经济一体化趋势持续深化，国际经济正在进入战后第三次产业大循环，世界范围的资本和产业转移加快，为我市承接国际转移、加快发展提供了更广阔的空间；综观国内，我国经济发展进入新一轮增长期，落实科学发展观和构建和谐社会进入良性轨道，社会主义新农村建设深入展开，为我市统筹城乡经济社会发展提供了难得机遇；着眼省内，区域协调发展战略深入实施，山区基础设施和产业发展得到更大支持，泛珠三角区域合作纵深推进，产业融合和产业升级调整步伐加快，为我市跨越式发展注入了强劲的动力；立足我市，区位交通条件极大改善，生态资源禀赋优势凸显，经济规模和产业基础增强，服务效率和社会管理水平显著提高，投资环境逐步优化，加快发展的承载力不断提高，已具备了推动经济社会实现跨越式发展的良好条件。这些有利条件正在加速聚合。但是，我们也必须清醒认识到，国际国内的竞争日趋激烈，我市面临的竞争压力加剧。特别是我市人均GDP突破1000美元之后，经济社会发展处于一个新的历史时期，如何进一步扩大经济发展规模、提高经济发展质量、增强经济发展后劲，实现新的跨越发展，这是摆在我们面前的新的重大课题。为此，我们必须认清形势，乘胜前进，以更大的勇气和决心，再创我市经济社会发展的新辉煌。

今后五年工作的总体要求是：高举邓小平理论和“三个代表"重要思想伟大旗帜，以科学发展观统领经济社会发展全局，紧紧围绕市第五次党代会确定的奋斗目标和总体部署，在更高层次上实施“三化一园”发展战略。坚持提升发展质量效益，更加注重产业优化升级，促进经济增长方式转变，实现经济又好又快发展；坚持城乡区域统筹发展，更加注重发展县域经济、民族地区经济和民营经济，抓好社会主义新农村建设；坚持经济社会协调发展，更加注重发展社会事业，大力推动文明城市创建，提高城市形象和市民素质；坚持经济社会可持续发展，更加注重环保和节能，建设绿色生态清远，构建资源节约、环境友好型社会；坚持依法行政、高效廉政，更加注重民主、法制建设，营造良好投资环境，提高对外开放水平；坚持以人为本、关注民生，更加注重解决人民群众最关心、最直接、最现实的利益问题，扎实推进和谐清远建设，为建设全面小康社会，基本实现现代化奠定更加坚实的基础。

今后五年的奋斗目标是：全面实现山区发展排头兵的目标，建设科学发展、快速发展、协调发展、和谐发展的新清远。

――经济基础更加坚实。全市生产总值突破1000亿元，比2006年翻一番多，年均增长18%，人均GDP超过全国平均水平；固定资产投资年均增长20%；地方财政收入年均增长20%；社会消费品零售总额年均增长16%，外贸出口总额年均增长25%，实际利用外资年均增长25%。经济发展的自主性、稳定性和自主创新能力明显增强，经济发展质量明显提高。经济结构不断优化。新型工业加快发展，主导地位更加明显；服务业水平提高，服务档次提升；全面推进社会主义新农村建设，现代农业稳步发展，完成农村经济“八个五”工程建设任务，实现种养比例调头，农民收入稳步提高，生产生活环境更为舒适。加快转变经济增长方式，促进产业结构优化升级，产业内部结构更加合理。资源节约型和环境友好型社会建设明显进步，单位生产总值能耗比2006年末降低16%以上。

――基础设施更加完善。加快推进交通网络建设，全面完成第二个“八路一桥”建设任务，启动和推进第三个“八路一桥”工程建设，到2011年，公路通车里程达到1.3万公里，每百平方公里公路密度达到68公里；完成500 公里高速公路建设和改造任务，实现市到县通高速或一级公路，县到乡镇通三级以上公路，乡镇到村委会通等级公路，全面完成乡村道路硬化建设任务；积极推进北江航道整治及岸线码头建设，形成连接珠三角的较为安全、便捷和完善的综合交通体系。加大通信建设改造力度，争取进入全省中上水平。全面加强水利建设，完成城乡水利防灾减灾建设工程、机电排灌工程和农村饮水安全五年规划建设任务，防洪抗旱能力明显增强。加快电源电网建设，推进抽水蓄能电站、500千伏贤令山输变电工程、粤连电厂及阳山电厂建设改造，争取启动煤矸石、核能发电项目建设，基本解决电力供给问题。

――城市功能更加优化。创建“中国宜居城市”，完成飞来湖、大燕湖工程建设，改善北江、大燕河、笔架河生态环境，基本建成清远水利枢纽工程，共同奠定“湖城”、“水城”框架。完成清州大道等十条市政道路建设，城市空间更加优化。启动城市经济适用房和廉租房工程建设；进一步完善城市服务配套设施，城镇人均公共绿地面积达到12平方米，城市亮化率达到98%以上,城镇生活污水处理率达到55%，环保综合指标达到87分, 城市美化、绿化、净化水平进一步提高，城市功能更加完善。加快县城和22个中心镇建设、改造步伐，提升城镇品位，增强人口聚居能力，全市城市化水平达到50%以上，城市生态更加优美，人居环境更加舒适。

――社会发展更加和谐。加大各项社会事业投入。建立教育事业投入长效机制，未来五年，国家、省、市、县等各级财政将投入81.3亿元,比前四年增加40.7亿元，全面提升基础教育、职业教育、高等教育办学质量。基本普及高中和中职教育，毛入学率要达到80%；高质量、高标准巩固九年义务教育，免收农村初中、小学学生的学杂费、书本费，让农村适龄少年儿童不再因贫困而失学、辍学。建立卫生事业投入长效机制，各级财政将投入16.6亿元,比前四年增加9.2亿元，加快公共卫生、农村卫生、社区卫生和农村合作医疗事业发展，健全公共卫生体系，全市农村合作医疗要稳定在95%以上，做到人人享有初级卫生保健，基本解决人民群众看病难、看病贵、因病返贫的问题。创建全国“双拥模范城市”和“文明城市”，进一步增强军政军民团结，提升城乡文明程度和公民思想道德素质。

――人民生活更加殷实。在岗职工年人均工资年均增长10%左右，基本达到全省中等水平；农民年人均纯收入年均增长8%左右，过上更加富裕的小康生活。人口自然增长率控制在6‰以内。社会就业更加充分，社会保障更加完善，救助体系更加健全。未来五年，抚恤和社会救济将安排12.1亿元,比前四年增加6.8亿元,切实帮助弱势群体解决生产生活问题，城乡最低生活保障按照省制定的城市每人每月80元、农村每人每月40元的补贴标准实施，努力让全市人民老有所养、病有所医、弱有所助、贫有所济，普遍得到更多实惠，过上更加富足的生活。

――政府效能更加提升。政府经济管理和社会管理不断科学、规范，依法行政水平不断提高，调控能力不断增强，运行机制和监督机制进一步健全，民主法制建设进一步加强，营造政通人和、干事创业的良好环境。城乡稳定，社会秩序良好，人的权益得到切实尊重和保障，全社会形成和睦相助、友爱向善的良好氛围，全体市民和谐相处，诚信感、安全感、幸福感进一步增强。

实现上述目标和要求，必须处理好四个关系：一是要处理好经济发展与生态环境保护及资源可持续利用的关系。要在加快经济发展的同时,加强对自然环境的保护,大力发展循环经济,提高资源利用的经济、社会和生态效益；二是要处理好经济发展与社会发展的关系。要加强经济社会发展中的薄弱环节,把资源引导到目前相对薄弱的社会发展、农业生产等方面,加强能源、交通、水利和城乡公共设施建设；三是要处理好地区之间的协调发展与互动的关系。要按照“五个统筹”要求，创造公平、合理的运行机制，加大对北部地区，尤其是少数民族地区的倾斜、支持力度，促进区域协调发展；四是要处理好内源型发展和外源型发展的关系。要大力推动民营经济发展，提高招商引资质量。

2007年工作安排

2007年，是新一届政府的开局之年，做好各项工作，影响长远，意义重大。按照“创新年”、“教育年”和“环保年”的总体要求，今年政府工作要：围绕“一个目标”、突出“两大主题”、推动“三项重点工作”。

围绕“一个目标”：就是争当山区发展排头兵。

突出“两大主题”：一是突出落实科学发展观。坚持五个统筹，努力实现速度、质量、效益相协调，消费、投资、出口相协调，人口、资源、环境相协调，提高发展的协调性、全面性与可持续性；二是突出构建和谐社会。进一步加强社会管理，更加关注民生，着力解决人民群众最关心、最直接、最现实的利益问题，促进公平正义，切实维护社会稳定，让改革发展成果惠及全市人民，加快和谐清远建设。

推动“三项重点工作”：一是推动创新。加快形成有利于经济社会科学发展、和谐发展的体制机制，加快创新型社会建设；二是加快教育发展。大力优化教育资源，加强素质教育，提高职业教育水平和“普九”质量，积极促进教育公平，推动教育等社会各项事业全面发展；三是依法加强环境整治和生态保护。推动资源节约型和环境友好型社会建设。

今年全市经济社会发展的主要预期目标是：生产总值增长18%，其中，一、二、三产业分别增长4%、26%和15%；单位生产总值能耗下降3.5%；地方财政一般预算收入增长20%；全社会固定资产投资增长20%；实际利用外资增长25%；社会消费品零售总额增长18%；外贸出口总额增长25%；居民消费品价格指数控制在3%以内；在岗职工年人均工资增长12%；农村居民人均年纯收入增长8%；城镇登记失业率控制在3.2%左右；人口自然增长率控制在6‰以下。

为实现今年经济社会发展预期目标，必须做好以下十项工作：

一、落实科学发展观，力促经济又好又快发展

推动工业结构优化升级，打造优势产业集群。培育支柱产业群，做大做强建材、机械、冶金、电子、化工等支柱产业。充分发挥资源优势，因地制宜发展资源型工业，扩大产业链。大力发展水泥、陶瓷、有色金属、电子等主导产业；培育战略产业，实现高新技术产业跨越式发展，积极发展中医药、新材料、新能源等高新技术产业。规范发展再生资源行业。抓紧规划建设中国（清远）循环经济产业园，探索清洁生产和资源综合利用的新路子，打造全国铜、铝、铁深加工基地。大力发展规模经济。发展特色产业集群，拉长产业链条，培育骨干企业。促进龙头企业和名牌产品的规模生产，依托主导产品，大力发展配套企业，增强产业配套能力。运用高新技术和先进适用技术改造提升传统产业，加快淘汰落后工艺技术，依法关闭高污染、高消耗、低效益的企业。

优化产业结构布局，促进区域协调发展。一是促进中、南部产业集群发展，增强产业自我扩张能力。加快水泥、陶瓷、金属拆解、制鞋等产业集群发展，引进和扶持一批大企业，延伸、完善产业链，形成特色鲜明的产业集群，增强产业竞争力。二是加大北部地区资源集约开发力度，促进北部地区经济崛起。坚持用办特区的理念发展少数民族地区经济，加大对北部山区，尤其是少数民族地区基础设施建设和公共服务的支持力度，提高承接产业转移能力。加强北部山区土地、矿产、水利、生物等资源的集约开发，重点发展生态、民族特色旅游业、资源加工业、清洁能源工业、医药、食品加工业等特色产业。加强南北经济合作，推进互补型经济发展，逐步缩小与南部地区的差距。

做大做强内源型经济，增强县域经济发展能力。加快构建民营经济综合服务体系，为民营企业搭建“展示、交流、交易、合作”平台，为民营经济发展创造更加良好的发展环境，不断壮大民营经济。因地制宜，大力发展农副产品加工业、资源加工业、配套工业和农产品流通业，继续做大做强现有十亿元以上乡镇，培育一批新的十亿元乡镇，加快县域经济发展。

积极实施名牌带动战略，提升产业竞争力。鼓励和支持各类企业特别是重点企业争创全国、全省名牌产品，国家级、省级著名商标，国家免 检产品。发展壮大现有名牌产品，逐步打造品牌产业群。依托特色产业和专业镇，培育和发展一批区域品牌。

二、进一步推进改革创新，打造新的发展动力

夯实自主创新基础。深化科技体制改革，整合科技资源，完善自主创新激励机制。落实科技三项经费的投入，加快市专业技术创新试点镇建设。进一步建立健全区域技术创新和特色产业信息化技术服务平台，为中小企业提供科技服务和技术支持。启动国家水泥检测中心建设工作，突出抓好以新北江企业博士后工作站、新亚光电缆公司等重点大型企业为主体的技术创新体系建设，引导企业在加强引进的同时，加大对科研、技术的投入力度，改造、提升和开发新的技术设备，提高原始创新和引进消化吸收再创新能力，促进产品由低端向高端提升，不断增强企业和产品竞争力。加快清远高新技术产业开发区建设，尤其是高新区“孵化器”的建设，使之成为我市技术创新、人才引进和培养、改造和提升传统产业的基地。加强与高等院校和科研院所的产学研合作，加快科技成果的转化与应用。

加大体制创新力度。加大知识产权保护和科技创新宣传力度。继续推进行政管理体制改革，推动政府职能转变，不断强化政府履行经济调节、市场监管职能。深化人事制度改革，积极稳妥开展事业单位改革试点工作。继续深化行政审批制度改革，加快建设行政审批电子监察系统，完善行政审批监管机制，规范行政审批行为。创新投资体制，进一步开放投资领域，鼓励民资和外资进入基础设施、教育、卫生等公共事业，吸引民间和外来投资参与我市各项建设。全面完成国有企业改革任务，创新国有资产监管体制，促进国有企业资产优化重组，提高国有资本运营效益。

三、发挥园区集聚效应，提升招商引资水平

抓好园区的规划与建设，提升园区档次。加快产业转移园区建设步伐，并在有条件的县（市）设立分园，将产业转移园区建设与发展产业集群有机结合起来，推动产业的更大集聚和优化升级。进一步整合高新技术产业开发区资源，加快中心区域老工业园区改造，提高园区集约开发水平。加快完善园区道路、水电、通讯等基础设施建设，提升园区美化、绿化、亮化水平，打造集经济、技术、绿色于一体的品牌园区，使之成为全市招商引资的窗口，经济发展的引擎。完善园区公交线路，加强对园区文化、卫生、娱乐等生活配套设施的规划、建设指导，增强园区人口集聚能力。

创新招商引资方式，提高招商引资质量。继续实施招商引资方式的“四个转变”，实行有选择招商。积极鼓励外商在现有基础上增资扩产，做大做强。重点抓好大项目的引进，加大对发达国家、地区和跨国公司、大企业，尤其是世界500强、技术密集型和资源加工型企业招商引资力度。优化吸收外资结构，加强对外商投资区域和产业的引导，吸引外资投向服务行业、特色产业、配套产业，引导资源加工型和劳动密集型产业向北部山区转移。

加强区域经贸合作，不断拓展发展空间。积极探索加强和深化与省内、国内乃至国际经贸合作的有效途径，积极有效承接珠三角等国内外产业转移，拓宽合作领域，提高合作层次。

四、加强“三农”工作，推动社会主义新农村建设

抓好新农村规划与建设。按照因地制宜、突出特色的原则，科学编制社会主义新农村建设总体规划和村庄规划及产业规划，着力推进80个示范村建设。加大经济薄弱村帮扶力度，继续抓好农村“五改”工作。市级财政今年安排1000万元用于新农村及农村安居工程建设，同时广泛发动群众及社会力量参与推动新农村建设。

提升农业产业化水平。加强农业科技创新和服务体系建设，加大农业先进技术推广应用，积极推进农业机械化。进一步加强清西现代农业示范区建设，突出抓好种源工程建设，加快推广沼气应用。推进农业标准化、基地化、品牌化生产，继续抓好农村经济“八个五”工程建设，全面落实各项支农惠农政策措施，积极推进名优家禽、沙糖桔、有机稻、无公害蔬菜、奶牛等“五大特色产业带”以及优质烟叶、中药材、优质竹笋等“三大基地”建设。举办好华南（清远）第三届农业博览会，打造现代农业发展平台。加快品牌农业和特色农业发展，进一步提升清远鸡、天农鸭、乌鬃鹅、温氏猪、英德奶牛等农业品牌效应。今年市级财政安排300万元，大力推进农村“一乡一品”建设，加快龙头企业的引进与培植，形成具有竞争优势的农产品加工企业集群。进一步加强引导和服务，继续探索农户与龙头企业利益共享、风险共担的联接机制，增强产业化经营发展后劲，切实加快发展现代农业。

强化农产品质量安全监管。启动并加快各县（市、区）农产品质量检测中心建设，切实抓好农产品质量安全工作，确保人民群众吃上放心肉、放心菜。加强对高致病性禽流感等重大动植物疫情的防控。

继续加大劳务输出力度。建立和完善劳动力市场需求信息网络，努力提高劳务经济的组织化程度。完善政策，为发展劳务经济提供强有力支持。继续引导农民向二、三产业转移，完成劳务输出10万人任务，不断提高农民收入。

五、大力发展现代服务业，推动第三产业上新台阶

加快发展旅游业。实施旅游旺市战略，全力打造旅游强市。做好旅游发展总体规划的评审和实施，抓好景区景点、旅游服务中心、五星级旅游接待酒店的建设，完善各项配套设施。大力开拓商务、城郊及“农家乐”旅游项目，打造更多国家4A级旅游风景区。加大对外宣传力度，打造“自然生态”、“中国奇洞之乡”等新品牌，争取更多的县（市）创建“中国优秀旅游城市”或国家级、省级旅游强县。

加快发展流通业。大力促进连锁经营、现代物流、中高级批发市场和电子商务等现代流通方式的发展，促进流通业结构调整和优化升级。优化农产品流通市场，大力发展农村经纪人和运销大户，加快产地和城郊农产品批发市场建设，促进农产品流通。加快农村流通网络体系建设，建立城乡一体化流通体系，稳步推进“万村千乡”市场工程试点，扩大农村消费。

加快专业市场的规划与建设。加快有色金属、建材陶瓷、汽车配件、五金家电等专业批发市场建设，扶持建设一批各具潜力的集散市场以及规模化、集约化、专业化的批发市场。

积极发展其他服务业。规范发展房地产业。打破行业垄断，规范市场准入，引进先进经营理念、服务手段和技术，加快金融、中介、会展、设计、信息咨询等现代服务业的发展，促进服务业与制造业良性互动发展。

六、加大基础设施建设力度，不断改善生产生活条件

今年，全市新安排重点建设项目135个，计划总投资629.42亿元，年度投资209.61亿元。

进一步完善交通基础设施。加快推进第二个“八路一桥”工程建设，清连公路高速化工程完成路面改造任务，其余项目力争基本完成。加快启动第三个“八路一桥”重点工程建设项目。积极推动武广铁路客运专线、二广高速和清州大道建设，加快京珠高速复线粤境段、广清高速清远段扩建和银英公路二级改造等工程建设前期工作。加快行政村公路硬化步伐，加大公路运输站场及大、小北江航道建设力度，提高综合交通运输水平。

加强水利设施建设。加快推进清远水利枢纽工程建设。确保28宗城乡水利防灾减灾工程按期完成，做好清城联围、清东围、茶坑水库等工程的竣工验收工作。加大对农田水利和节水灌溉工程建设投入，继续抓好小型水库除险加固、贫困山区农村机电排灌工程、农村小水电、农村饮水安全等工程建设，做好水库移民遗留问题议案实施工作。

加大能源建设力度。加强电力设施建设，加快220千伏朗新、燕河、浩源输变电工程项目建设，抓好贤令山500千伏和石角、连山220千伏输变电工程前期工作，争取尽早动工建设。加强协调与配合，加快抽水蓄能电站建设。继续争取核能发电、“以大带小”煤电、煤矸石发电、粤连火电三期立项，继续抓好小水电挖潜改造工作。

加快市政基础设施建设。加快创建“中国宜居城市”步伐，着力建设宜居宜业的健康城市，让市民最大限度地分享城市建设的成果，保持身心健康，尽情享受生活。抓好清远大道延伸线、连江南路等市政道路的改造和建设，使市区主干道路基本实现“黑底化”和路灯普及。继续加大城中村整治改造力度。基本完成飞来湖挖湖、护坡及吸排水工程建设，启动并加快推进大燕湖以及中轴线、环城水系建设。完成市区一批公厕的改造与建设规划，加大中山公园的整治改造力度，进一步提升城市绿化、净化、亮化水平。各县（市）要抓好城区和中心镇市政基础设施建设，完善配套功能，提高人口、产业聚集能力，带动区域经济发展。

推动信息化建设。加大投入力度，推进宽带通信网、数字电视网等信息基础网络建设，提高网络功能和覆盖面。推进党政内网和电子政务应用系统建设，推进社会公共服务领域信息化。

七、抓好财税金融工作，增强发展保障能力

继续完善公共财政体制。深化国库集中支付、部门预算、政府采购、财政支出绩效评价等财政改革，推动公共财政建设。进一步调整财政支出结构，加大财政在“三农”、教育、卫生、科技、文化、就业再就业、社会保障、生态环境、公共基础设施、社会治安等方面的投入。加强财政监督，提高财政资金使用效益。深化收支两条线管理，建立健全行政事业单位福利机制。

进一步强化税收征管。加强税源监控，强化对重点税源、重点行业的税收管理，大力组织收入。进一步完善护税协税网络，构建融洽的税企关系。加强信息化建设，提高纳税服务水平。坚持依法治税，严厉打击各种偷、逃、骗税行为，堵塞征管漏洞。在确保应征尽收的同时，决不收过头税，保证我市财税持续稳定健康发展。

建设良好金融生态。加快信用体系建设，保护金融债权。创新机制，发挥金融对经济发展的支撑作用，加快解决中小企业和民营企业融资难问题。深化农信社改革，发挥好其支农的主导作用。加强产品创新，不断优化保险结构。

八、坚持优先发展教育，提高全民素质

加大教育投入力度，建立教育投入长效机制，今年各级财政将安排13.3亿元支持教育发展。其中市直财政安排8810万元，比上年增加4800万元，重点解决学校建设债务、教师补贴以及教师定编问题。加大公共教育资源向农村、贫困山区、少数民族地区的倾斜力度，逐步缩小城乡、区域教育发展差距。继续推进农村义务教育经费保障机制改革，鼓励社会捐资助学，保障家庭经济困难学生接受教育。全面落实农村免费义务教育政策，从今年秋季起再免收农村义务教育阶段书本费，实现完全意义上的农村免费义务教育。认真解决非户籍常住人口子女义务教育问题，不断巩固“普九”成果。深化教育改革，优化教育结构，大力实施素质教育，进一步加大教育资源整合力度，大力推进义务教育学校规范化建设。继续加快高中和民办教育的发展，大力发展职业技术教育，重点抓好南华工商学院、市一中实验学校二期工程、新华侨中学、清远职业技术学院二期、清远市高级技工学校（技师学院）扩建二期工程以及省批准的7所骨干示范性中等职业学校的建设，打造教育品牌。加强教育收费项目和标准督查，坚决制止乱收费。规范办学行为，引导民办教育健康发展。大力实施“强师兴教”工程，提高师资特别是农村师资水平。全面推进教育基础网的建设与普及应用。继续开展教育强镇创建工作。

九、加强资源节约和生态环境建设，促进可持续发展

认真落实节能降耗和减少污染排放目标责任制，严格环保责任考核制度。今年市级财政安排环保专项资金500万元，以解决危害群众健康和影响可持续发展的环境问题为重点，加强对水源、森林等自然资源的保护，加大对重点污染源的整治。加快自然生态区（市肺）工程建设，继续抓好创建林业生态县工作。加快城市、工业园区污水处理厂、生活垃圾及固体废物处理设施建设，清理、整顿市区废品收购站场，逐步实现废品定点收购。加快循环产业园区建设，大力发展循环经济。突出抓好工业废水排放大户和重点污染行业的治理，继续在水泥、陶瓷、有色金属、化工等重点行业推行清洁生产。加大村容村貌整治力度，加强农业农村环境污染治理。加快建立有利于促进节能增效的市场调节机制和政策措施，广泛开展节能节水节地节材活动，促进节能增效。坚决落实“三个一律”原则，严格控制建设占地规模，进一步盘活存量土地，重点消化利用各类开发区的闲置土地，提高土地利用效率。严格环保市场准入，从源头上控制污染。严格环境执法，严肃处理环境违法行为。加强环保教育，大力倡导健康、节约、环保的消费模式和良好社会风气，提高全社会节约资源、保护环境意识。

十、坚持以人为本，构建和谐新清远

实施便民廉医工程。加大医疗卫生事业投入，建立卫生事业投入长效机制，今年各级财政将安排2.7亿元，其中市直财政安排7539万元，支持医疗卫生事业。市直财政安排2300万元，比上年增加1670万元，扶持农村合作医疗，争取新型农村合作医疗参保率达到95%以上，积极实施“每村一个卫生站、一名医生”建设，争取做到小病不出村，逐步解决群众看病难问题。完善公共卫生体系和卫生应急机制，进一步提高疾病预防控制和卫生应急能力。加强学校、企业集体食堂和公共餐饮单位的卫生监督管理，保障人民健康安全。继续加强重大传染病的监测，做好非典型性肺炎、人禽流感等的防控及艾滋病、慢性病、地方病、职业病等的防治工作。积极开展农村初级卫生保健，加强城市社区卫生基础建设。开展爱国卫生运动。发展妇幼卫生事业。加快市人民医院新院区、市慢病院疗养分院及基层医疗卫生设施建设。加强各级中医院建设，推进中医药事业发展。加强行风和医德医风建设，建立治理医药购销领域商业贿赂长效机制。

着力改善民生。扩大城乡最低生活保障覆盖面，农村低保覆盖率力争达到100%；提高城乡最低生活保障水平，按照省制定的补贴标准实施，即城市每人每月补贴80元，农村每人每月补贴40元，分别比上年提高53.8%和60%。继续加大社会保险的扩面征缴、清欠力度，完善养老、医疗、失业、工伤和生育保险制度。推进企业职工基本养老保险制度建设，吸引非公有制企业和灵活就业人员参保，进一步扩大社会保障覆盖面。全面理顺县级劳动保障和社会保障体制，进一步完善社会保险市级统筹工作机制。加强基金监管，加快解决社会保险历史遗留问题。今年各级财政将安排2亿元用于以优抚安置、扶老、助残、救孤、济困为重点的慈善事业和社会福利事业。实施全民安居工程，抓好农村安居工程，今年底解决贫困户住房困难问题；加快推进经济适用房和廉租房建设，解决城镇特困户和中低收入家庭住房难问题。发展残疾人事业，保障残疾人合法权益。创新扶贫工作思路，大力推进科技、教育和智力扶贫。坚持就业优先原则，创新城乡统筹就业制度，加大公益性岗位开发力度，加强对大学毕业生的就业指导和服务，大力推进富余劳动力转移就业。开展“双无”（无双下岗或双失业社区）等就业援助专项行动，加强对零就业家庭、“4050”人员和农村贫困家庭的再就业援助，让弱势群体有更多的关爱，让千家万户有更多的笑容，让困难群众有更多的温暖。加强职业技能培训，全面实施“广东省百万农村青年技能培训工程”和退役士兵免费职业技能培训工作。进一步完善劳动关系调整机制，维护劳动者合法权益，构建和谐稳定的劳动关系。

着力抓好安全生产。加强监管，防止重大事故、杜绝特大事故发生，力争各类安全事故死亡人数比上年下降3%。建立健全安全生产目标责任体系，强化安全生产责任制考核，严格安全执法。实行安全准入，建立经济约束机制。加大安全生产专项整治，突出抓好道路交通、非煤矿山、危险化学品、民爆物品、“三小”单位和人员密集场所消防安全以及建筑施工、城市燃气、农用车非法载客、特种设备等安全专项整治，进一步巩固煤矿安全专项整治成果。建立、完善应急救援体系，切实加强队伍建设，提高事故应变能力。

着力维护社会稳定。加快社会治安防控体系建设，着力整治突出治安问题，严厉打击各种黑恶势力、严重刑事犯罪和“两抢”、“两盗”等多发性犯罪，坚决扫除“黄赌毒”、传销和变相传销及“六合彩”赌博。大力开展和谐社区建设，把社会治安综合治理的各项措施落实到基层，提高群众安全感。继续整顿和规范市场秩序，依法严厉打击假冒伪劣、价格欺诈等不法行为，深入治理商业贿赂，营造安全可靠的消费环境。加强信访和基层人民调解工作，统筹协调各方利益关系，加强矛盾纠纷排查调处，有效预防和化解各种社会矛盾，强化群体性事件预防处置机制，提高应对突发事件的能力。

着力推进社会各项事业全面发展。深入开展“两无”工作（镇街无政策外多孩生育，村居无政策外出生），巩固人口与计划生育工作成果，加大综合治理出生人口性别比升高力度，提高人口素质。进一步深化文化体制改革，加快建立政府主导、社会参与兴办文化事业的格局。启动创建全国文明城市工作，加强公益性文化设施建设，加快博物馆、图书馆、科技馆和群众艺术馆等馆场的资源整合，做到物尽其用。加强对文化遗产的挖掘、整理、保护与利用，申报一批国家、省级文物保护单位。加快广播电视村村通工程建设，消灭电视覆盖盲点。取缔违法网吧、游戏机室，营造文明和谐的文化市场秩序。积极发展体育事业，加快筹建龙舟竞赛基地和天然泳场，加大申报“国家足球训练之乡”力度，做大做强生态体育品牌。抓好文化体育公园的规划，加快城乡社区体育设施建设，广泛开展全民健身活动。加强公民思想道德建设，开展以“八荣八耻”为主要内容的社会主义荣辱观教育。加强少数民族工作，促进民族团结。进一步加强全民国防教育和民兵预备役工作。继续做好统计、审计、物价、外事、侨务、对台、人事、妇女儿童、宗教、扶贫、移民、人防、气象、地震、档案、方志、殡改等各项工作。

加强政府自身建设

新一届政府肩负着继往开来，与时俱进的历史重任，承载着全市人民的重托。做好新形势下的政府工作，对于如期实现未来五年的发展目标，推动全市经济社会更好更快地发展具有十分重要的意义。新一届政府将按照“为民、务实、清廉”的要求，把全心全意为人民服务作为政府的根本宗旨，把不断满足人民日益增长的物质文化需求作为政府的根本任务，把人民满意不满意、拥护不拥护、赞成不赞成作为衡量政府工作的根本标准，全面加强政府自身建设，不断推进政府管理创新，努力加快法治政府、服务政府、责任政府、效能政府、廉洁政府建设进程，真正做到为人民服务，对人民负责，受人民监督，让人民满意。

一、不断加强学习，以能力促提高

面对新的形势和任务，面对新的机遇和挑战，政府各部门及其工作人员必须把学习作为一种政治责任和精神追求，牢固树立终身学习和创造性学习的理念，把学习的过程转化为研究问题、解决问题的过程，不断增强政治敏锐性和政治鉴别力，做到不迷失方向，不偏离中心。不断增强统揽全局和科学决策的能力，做到指导工作不违背规律，推动工作不脱离实际。大兴调查研究之风，把发展的热情同科学的态度和务实的作风统一起来，把各项决策建立在科学、民主、法制的基础之上，使各项决策经得起实践的检验，历史的检验。发展时不我待，机遇稍纵即逝。我们必须进一步增强不进则退、小进也是退的危机感和责无旁贷、义不容辞的责任感，以振兴清远为己任，在挑战中寻求机遇，在问题中寻求办法，在困难中寻找出路。善于用开放的意识正视存在的差距，用超前的理念研究争先的思路，用积极的办法落实发展的措施，聚精会神搞建设，一心一意谋发展，努力使清远一年一个样、五年大变样。

二、加快职能转变，以高效促服务

坚持有所为有所不为，进一步转变政府职能，使政府职能真正转到宏观调控、市场监管、社会管理和公共服务上来。进一步减少和规范行政审批事项，积极推进政务公开，不断提高政府工作透明度。继续抓好行政服务中心建设，全面落实一站式办公、承诺服务等工作机制，规范行政行为，切实履行好为企业服务、为纳税人服务、为创造财富的人服务的行政职能。要敢于吃苦耐劳，乐于奉献，着力解决人民群众密切关注的热点、难点问题以及反映强烈的突出问题，全面优化政策环境、市场环境、法制环境和投资创业环境，把政策交给投资者，把方便留给创业者。时刻牢记党和人民的重托，牢记富民强市的重责，牢记跨越式发展的重任，扎扎实实干好本职工作。要进一步完善公务员考核激励机制，促进政府全体工作人员精心谋事、潜心干事、坦荡处事、尽职办事，做到权为民所用，情为民所系，利为民所谋。要改进工作作风，我们一切工作的出发点和归宿都是为了实现人民群众的利益，一个对群众怀有深厚感情的干部，不会对群众的冷暖疾苦漠然置之，不会在群众面前颐指气使，更不会动辄以强硬手段对待群众。各级领导干部要牢记党的宗旨，不忘掌权为了谁，发展为了谁，切实把人民群众的利益放在首位，真心实意为群众办实事、解难事、做好事，不断密切与群众的联系，增强在群众中的影响力。

三、坚持依法行政，以民主促和谐

坚持依法治市，加快政府工作法制化进程。增强政府部门领导干部和公务员的法制观念，提高依法行政水平。坚持依法治市和以德治市相结合，进一步扩大基层民主，确保群众依法行使民主选举、民主决策、民主管理和民主监督的权利。自觉接受市人大及其常委会的依法监督和市政协的民主监督，高质量办理人大代表、政协委员的建议、提案。加强与工商联、民主党派、无党派爱国人士的联系，主动听取工会、共青团、妇联等群团组织和社会各界的意见，集思广益，不断提高决策能力和依法行政水平。认真贯彻落实《行政许可法》和《公务员法》，进一步明确行政执法权限，完善政府部门工作制度，增强公务员社会主义法制理念，正确行使行政权力。全面落实行政执法责任制，切实把政府管理经济社会行为纳入依法运转的轨道，做到文明执法、规范执法、公正执法。

四、强化责任意识，以落实促工作

进一步完善重大问题集体决策、专家咨询、行政问责和督查督办等制度。建立以行政首长为重点的行政问责制度，把行政问责与行政监察、审计监督结合起来，加大对行政效能过错、失信失诺等案件的查办力度，严肃查处政令不畅、办事效率低下、自由裁量失当等问题，切实增强政府执行力和公信力。不断完善目标管理责任制，建立起奖罚分明的目标激励机制，加大对重点工作、重点项目的协调和督查力度，不断提高工作效率，确保各项工作落到实处。着力建设信用政府，诚信清远，加强社会诚信宣传教育，营造诚实守信的社会环境。严格依法办事程序，强化法律咨询服务，加强对合同契约的审核。

五、坚持从严治政，以廉洁塑形象

认真贯彻标本兼治、综合治理、惩防并举、注重预防的反腐倡廉工作方针，推进教育、制度、监督并重的惩治和预防腐败体系建设。完善项目公开招投标、工程基建项目跟踪审计、项目监理、项目法人责任等制度，预防和制止项目建设中的腐败现象。完善部门预算、政府采购、重大开支、财务公开、干部人事、行业自律和责任追究等各项制度，从源头上、制度上规范行政行为。大力开展治理商业贿赂专项工作，重点治理工程建设、土地出让、产权交易、医药购销和政府采购等领域的商业贿赂问题。坚持纠建并举的方针，切实纠正部门和行业不正之风，强化行政和审计监督，查处各类违纪违法案件，以卓有成效的反腐倡廉成果取信于民，树立良好的政府形象。所有政府工作人员都要树立正确的世界观、人生观和价值观，任何时候、任何情况，党的宗旨不能忘、不义之财不能取、自我约束不能松，始终做到自重、自省、自警、自励。要牢固树立过紧日子的思想，勤俭办一切事情，严禁铺张浪费，把有限的资金用在改善群众生活上，用在加快发展上。

各位代表，市第五次党代会绘就了清远未来五年发展的美好蓝图，历届班子的艰苦探索和大胆实践，为我们今后更好更快地发展奠定了坚实的基础，也积累了丰富的经验。回顾过去，我们心潮澎湃；展望未来，我们热血沸腾。争当山区发展排头兵，建设小康社会与和谐清远是我们奋斗的目标。让我们紧密团结在以胡锦涛同志为总书记的党中央周围，高举邓小平理论和“三个代表”重要思想伟大旗帜，坚持以科学发展观统领经济社会发展全局，在市委的正确领导下，抓住机遇，迎接挑战，同心同德，开拓创新，发奋图强，再铸辉煌，为实现美好蓝图而努力奋斗！
